# Bioavailability of transgenic microRNAs in genetically modified plants

**DOI:** 10.1186/s12263-017-0563-5

**Published:** 2017-07-07

**Authors:** Jian Yang, Cecilia Primo, Ismail Elbaz-Younes, Kendal D. Hirschi

**Affiliations:** 1 USDA/ARS Children’s Nutrition Research Center, Baylor College of Medicine, 1100 Bates Street, Houston, TX 77030 USA; 2 Vegetable and Fruit Improvement Center, Texas A&M University, College Station, TX 77845 USA

**Keywords:** Dietary microRNAs, Genetically modified organisms, Bioavailability, Digestive stability, MIR2911, Mice

## Abstract

**Background:**

Transgenic expression of small RNAs is a prevalent approach in agrobiotechnology for the global enhancement of plant foods. Meanwhile, emerging studies have, on the one hand, emphasized the potential of transgenic microRNAs (miRNAs) as novel dietary therapeutics and, on the other, suggested potential food safety issues if harmful miRNAs are absorbed and bioactive. For these reasons, it is necessary to evaluate the bioavailability of transgenic miRNAs in genetically modified crops.

**Results:**

As a pilot study, two transgenic Arabidopsis lines ectopically expressing unique miRNAs were compared and contrasted with the plant bioavailable small RNA MIR2911 for digestive stability and serum bioavailability. The expression levels of these transgenic miRNAs in Arabidopsis were found to be comparable to that of MIR2911 in fresh tissues. Assays of digestive stability in vitro and in vivo suggested the transgenic miRNAs and MIR2911 had comparable resistance to degradation. Healthy mice consuming diets rich in Arabidopsis lines expressing these miRNAs displayed MIR2911 in the bloodstream but no detectable levels of the transgenic miRNAs.

**Conclusions:**

These preliminary results imply digestive stability and high expression levels of miRNAs in plants do not readily equate to bioavailability. This initial work suggests novel engineering strategies be employed to enhance miRNA bioavailability when attempting to use transgenic foods as a delivery platform.

**Electronic supplementary material:**

The online version of this article (doi:10.1186/s12263-017-0563-5) contains supplementary material, which is available to authorized users.

## Background

Since 1996, genetically modified (GM) crops have been commonly consumed by the general public [1]. Excitement has also been generated that some of these GM plants can help alleviate diseases and malnutrition [2, 3]. The current safety assessments for these commercial crops predominately focus on the transgenic protein(s) [4, 5]. However, agrobiotechnology often expresses novel RNA molecules in crops. Both the safety and therapeutic potential of transgenic RNAs in GM crops should be thoughtfully addressed [6].

Both microRNAs (miRNAs) and small interfering RNAs (siRNAs) are classes of small RNAs (sRNAs) that regulate gene expression [7]. RNA interference (RNAi) is an umbrella term that defines conditions where a sRNA directs sequence-specific gene repression. The prevailing view is that dietary sRNAs are not absorbed by consumers [8–13]; however, recent studies suggest that consumers may absorb and circulate dietary RNAs [14–19]. The uptake of GM diet-derived RNAs in controlled animal feeding studies should be carefully examined in order to establish guidelines for risk assessment and therapeutic applications.

Bioavailability is defined as the portion of a substance that reaches systemic circulation, and the bioavailability of dietary sRNAs appears to be low [13, 20, 21]. However, sRNAs ingested from plant-based foods may act as potent bioactives and have been implicated in reversing specific diseases in several cases [15–18, 20, 22, 23]. Meanwhile, numerous groups have found that dietary sRNAs are not bioavailable in animals [8, 9, 13]. The ability/inability to detect these dietary sRNAs in consumers has been attributed to a variety of potential differences among research groups, including methodology inconsistencies, contamination, and detection sensitivity issues [20, 24–26]. To date, research labs that have successfully demonstrated serum uptake of plant-based sRNAs have not addressed bioavailability of transgenic miRNAs in plant-based diets. One of the concerns of GM crops is the introduction of foreign genes into crops and its unpredictable consequences. For example, it is theoretically possible that transgenic miRNAs possess unique chemical modifications which allow them to be more bioavailable than a native miRNA. As a result, the characterization of the bioavailability of transgenic foreign miRNAs is warranted.

Our group has successfully demonstrated that the plant-based sRNA MIR2911, found in a melody of vegetables, is more stable during digestion and is bioavailable to mice fed vegetable-based diets [14, 21, 27, 28]. Here we report the characterization of the digestive stability and dietary uptake of two miRNAs that are expressed in transgenic plants at levels comparable to the MIR2911 in fresh tissues. This small sampling of Arabidopsis lines suggests that transgenic miRNAs may not be readily bioavailable.

## Methods

### Generation of transgenic plant lines

The transgenic Arabidopsis line expressing the artificial miRNA termed amiR-RICE sequence (5′-UUU GGA AGC AAA GAA GCG GUG -3′) was obtained from Dr. Xiuren Zhang (Zhang et al.; personal communication 2015). The binary construct for overexpression of a murine miRNA mmu-miR146a (5′-UGA GAA CUG AAU UCC AUG GGU U -3′) in Arabidopsis was made using the Gateway system (Invitrogen, Carlsbad, CA). The destination vector pBA-DC [29] and template entry vector pENTR-amiR-CPC3-159a [29] were provided by Dr. Xiuren Zhang, and mmu-miR146a was cloned into the entry vector as previously described [29]. The sequences of the cloning primers which contained the incorporated mmu-miR146a sequences were as follows: forward-5′-AAG ATA GAT CTT GAT CTG ACG ATG GAA GAA CCT GTG AAA TTC AGT TCT TGC ATG AGT TGA GCA GGG TA -3′; reverse-5′-AAG ACC CGG GAT GAA CCC ATG GAA TTC AGT TCT CAG AAG AGT AAA AGC CAT TA -3′ (mmu-miR146a business and passenger strand sequences are underlined). The growth conditions of Arabidopsis line were as described [30]. Transgenic lines were distinguished from untransformed by BASTA selection. Homozygous lines harboring the transgenic mmu-miR146a constructs were selected by segregation analysis in the T3 generation. The lines were analyzed by qRT-PCR for mmu-miR146a expression, and the lines displaying the most robust shoot expression in adult plants grown on soil for 45 days (stage 6.50, https://www.arabidopsis.org/portals/education/growth.jsp) were used for diet preparation. From here on, the transgenic Arabidopsis lines used for mouse feeding studies and overexpressing amiR-RICE and mmu-miR146a are referred to as tg-RICE and tg-146, respectively.

### Plant diet preparation

The shoot tissues from approximately 45-day-old tg-RICE or tg-146 plants were harvested and freeze dried to 30% of fresh weight, and the dried tissues was then finely ground and mixed with finely ground chow (Teklad 2914, Envigo, UK) and water at 1:2:2 weight ratios, according to procedures described previously [14] and stored at −20 °C until use.

### Degradation of miRNAs in transgenic plant diets

After preparation, the diets were incubated at room temperature. A 10–20-mg fraction of diet was removed at 1, 4, and 24 h to assay the stability of miRNAs within the diet [31]. To isolate total RNA from the plant diet, the miRNEASY RNA isolation kit (Qiagen, Valencia, CA) was used according to the manufacturer’s instructions. Fifty femtomoles of an artificial miRNA termed C7 was used as an exogenous spike-in control.

### Animal feeding studies

The experimental protocol involving mice was approved by the Institutional Animal Care and Use Committee of Baylor College of Medicine. Specifically, the institutional animal protocols AN-2624, AN-6438, and AN-6454 cover the experiments performed in this study. All mice were obtained from the Center for Comparative Medicine at Baylor College of Medicine. Male ICR mice at 8 to 10 weeks old were used in all feeding studies, which were replicated at least three times; the results shown are representative of the biological replicates. Mice were fed with the plant diets for 3 days before they were sacrificed. Each day, 5 g of the plant diet that contained 1 g of dried plant material was fed to each mouse. The daily intake of plant material per mouse is equivalent to 3.3 g of fresh plant tissue.

### Serum collection and RNA extraction

Blood was collected via cardiac puncture as previously described [14]. Sera were separated at room temperature followed by centrifugation to remove all blood cells and debris. Total RNA was extracted from 100 μL of sera using the miRNEASY Kit following the manufacturer’s recommendations.

### Analysis of miRNA levels by qRT-PCR

TaqMan miRNA Assays for let-7dgi [32], C7, amiR-RICE, mmu-miR146a, MIR2911, and MIR168a were obtained from Life Technologies (Carlsbad, CA). Total RNA isolated was used in each reverse transcription (RT) reaction, as previously described [14]. To quantify miRNA levels in Arabidopsis, plant shoot material was ground to a fine powder in liquid nitrogen and then 10–20 mg was subjected to RNA isolation using the miRNeasy kit; 50 fmol of synthetic C7 was spiked into the plant Qiazol lysate as an exogenous RNA control. qRT-PCR was performed using a Biorad CFX96 Real-Time PCR Detection System, and data were analyzed using Biorad CFX software. Delta-Delta-Ct method was used to calculate the relative levels of miRNAs. Absolute concentrations of miRNAs were calculated based on standard curves obtained from serial dilutions of synthetic miRNAs [14] (Additional file 1: Figure S1).

### Preparation of synthetic miRNAs

Synthetic miRNAs were obtained from Integrated DNA Technologies (Coralville, IA) with 5′-phosphorylation and 2-O-methylation at the 3′ end nucleotide, to mimic the chemistry of plant-derived miRNAs. The sequence of the miRNAs were as follows: MIR-2911 5′-GGC CGG GGG ACG GGC UGG GA -3′; MIR-168a 5′-UCG CUU GGU GCA GAU CGG GAC -3′; amiR-RICE 5′-UUU GGA AGC AAA GAA GCG GUG -3′; mmu-miR146a 5′-UGA GAA CUG AAU UCC AUG GGU U -3′; C7 5′-GGA UCA UCU CAA GUC UUA CGU -3′; and MIR161 5′- UCA AUG CAU UGA AAG UGA CUA -3′. For gavage feeding, miRNAs were diluted in RNase-free phosphate-buffered saline (PBS), and each animal was fed 400 pmol of each miRNA in 300-μL volume [14].

### In vitro digestion of miRNAs with porcine enzymes

In vitro digestion conditions were as previously described [33, 34]. Briefly, the gastric phase was composed of a gastric electrolyte solution (7.8 mM K^+^, 72.2 mM Na^+^, 70.2 mM Cl^−^, 0.9 H2PO4^−^, 25.5 mM HCO3^−^, 0.1 mM Mg^2+^, 1.0 mM NH4^+^, 0.15 mM Ca^2+^) with pH adjusted by 1 N HCl to 2.0 and porcine pepsin (80 mg/mL) (Sigma, St. Louis, MO); the intestinal phase was formed by adding to the gastric phase an intestinal electrolyte solution (7.8 mM K^+^, 72.2 mM Na^+^, 124.4 mM Cl^−^, 55.5 H2PO4^−^, 85 mM HCO3^−^, 0.33 mM Mg^2+^, 0.6 mM Ca^2+^), 24 mg/mL of bile extract (Sigma, St. Louis, MO), and 40 mg/mL of porcine pancreatin (Sigma, St. Louis, MO) and 1 N NaOH to adjust the pH to 7.0. One-milliliter PBS solution containing 10 pmol each of MIR2911, C7, amiR-RICE, and mmu-miR146a synthetic miRNAs was first mixed with 1-mL gastric phase and digested with slow rotation at 37 °C for 60 min. The digestion mixture was then mixed with intestinal phase and digested with slow rotation at 37 °C for an additional 75 min. One hundred microliters of samples were removed at 30 min, 60 min of the gastric phase, and 5, 30, and 75 min of the intestinal phase for analysis of the levels of surviving miRNAs. One hundred microliters of pre-digestion samples were used as controls for calculating the percentage of surviving miRNAs. MIR161 was used as an exogenous spike-in control.

### In vivo digestion of gavaged miRNAs

ICR mice were fed purified diet (AIN-76a) for 7 days and then gavage-fed 400 pmol of MIR2911, C7, amiR-RICE, and mmu-miR146a. miRNA levels from the intestine were assessed 1 h post-gavage from the intestinal contents by flushing the excised intestines with 1 mL of PBS. One hundred microliters of the homogenized intestinal content was subject to RNA isolation and qRT-PCR analysis. MIR161 was used as an exogenous spike-in control.

### Assaying miRNA levels from diets in the small intestines

Mice fed transgenic diets and chow were used for this assay. The levels of miRNAs from the small intestine were determined using the intestinal contents collected by flushing the excised small intestines with 1 mL of PBS solution. One hundred microliters of the homogenized intestinal contents were analyzed by qRT-PCR for the levels of miRNAs.

### Statistical analysis

Statistical analyses were performed with the Student *t* test formula in Microsoft Excel. Significance was set at *p* < 0.05. Data were presented as means ± SEMs.

## Results

### Levels of transgenic miRNAs in plants

**Fig. 1:**
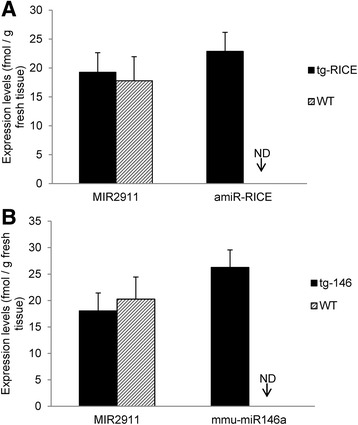
Quantification of engineered miRNA levels from transgenic plants. qRT-PCR quantification of the expression of amiR-RICE from transgenic plants (tg-RICE) (**a**) and mmu-miR146a from its transgenic plants (tg-146) (**b**) with the aerial shoot tissues of 45-day-old transgenic plants. *ND* none-detection. *N* = 3, *error bars* represent SEM

Arabidopsis was used for creating transgenic foods as it is a well-characterized model system [35]. Arabidopsis lines were engineered that express two miRNAs, amiR-RICE and mmu-miR146a. amiR-RICE is an artificial miRNA whose sequence has no homology to either plant or animal endogenous miRNAs, while mmu-miR146a’s sequence is identical to the endogenous murine miRNA [36]. The expression level of amiR-RICE lines has been characterized (Xiuren Zhang; personal communication 2015). For mmu-miR146a, plant lines that showed the most robust expression were used for further studies (Additional file 2: Figure S2). qRT-PCR quantification results demonstrated that the expression levels of amiR-RICE and mmu-miR146a reached levels of 22.9 and 26.3 fmol/g of fresh weight, respectively, which is similar to the levels of MIR2911 in fresh shoot tissues (approximately 18–19 fmol/g) (Fig. 1).

### Levels of transgenic miRNAs in diets

**Fig. 2:**
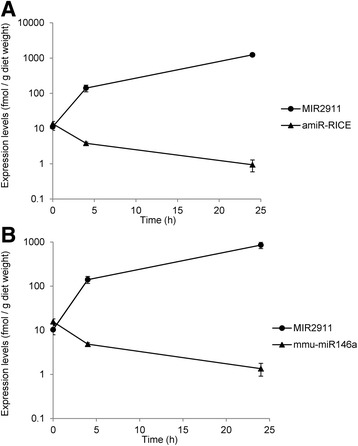
Transgenic miRNA stability in plant-based diets. Time course measurement of the levels of amiR-RICE (**a**) and mmu-miR146a (**b**) in comparison to MIR2911, in plant diets incubated at room temperature. Diet samples were assayed at 0, 4, and 24 h. *N* = 3, *error bars* represent SEM

Our previous studies demonstrate that dietary abundance of miRNAs can change during diet degradation and impact uptake in consumers [31]. Levels of amiR-RICE and mmu-miR146a were assayed to determine their stability relative to that of MIR2911, in the diets that were incubated at room temperature. The abundance of amiR-RICE and mmu-miR146a decreased gradually over time by approximately 10-fold, to approximately 1 fmol/g of diet, while MIR2911’s abundance, as we have demonstrated previously [31], was amplified by more than 85-fold after 24 h in the chow (Fig. 2).

### Digestive stability of transgenic miRNAs

**Fig. 3:**
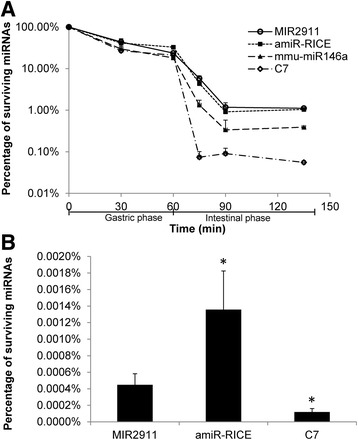
Stability of synthetic miRNAs during digestion. (**a**) Stability of amiR-RICE and mmu-miR146a, in comparison to MIR2911 and C7, in an in vitro porcine digestion assay. (**b**) in vivo assay of the digestive stability of the amiR-RICE, in comparison to MIR2911 and C7, in gavage-fed mice. The levels of surviving miRNAs in the small intestines were analyzed 1 h after feeding. *N* = 5, *error bars* represent SEM. *p < 0.05 between MIR2911 and amiR-RICE or C7

One of the main factors affecting bioavailability is the digestive stability of the nutrients [33]. Synthetic forms of the plant-based transgenic miRNAs, amiR-RICE and mmu-miR146a, were tested for their digestive stability using both an in vitro and in vivo assay. This was performed in comparison to MIR2911 and C7, which have been shown to have vastly different digestive stability in an in vitro assay, with MIR2911 being 10-fold more stable than C7 [27]. The in vitro digestion assay contained porcine digestion enzymes and demonstrated that amiR-RICE had similar digestive stability compared to MIR2911, while mmu-miR146a was significantly less stable than MIR2911, with surviving percentage in the intestinal phase after 75 min for mmu-miR146a being 0.39% comparing to 1.11% for MIR2911 (Fig. 3a). In the in vivo assay, gavage-fed synthetic miRNAs demonstrated much higher sensitivity to the murine digestive enzymes in vivo compared to the in vitro porcine system. After an hour, amiR-RICE had an approximately threefold higher survival percentage after gavage feeding compared to MIR2911 in the small intestines (0.0014% for amiR-RICE compared to 0.00045% for MIR2911) (Fig. 3b). Due to high background detection of endogenous mmu-miR146a in the murine intestines, the in vivo stability of mmu-miR146a was not analyzed (data not shown).

### Bioavailability of transgenic miRNAs

**Fig. 4:**
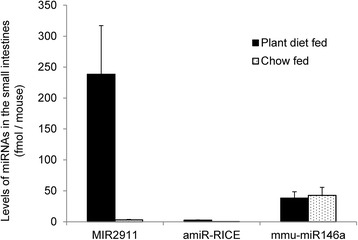
Levels of the transgenic miRNAs in the small intestines of mice fed a transgenic plant diet for 3 days. qRT-PCR quantification of levels of MIR2911 and the transgenic miRNAs amiR-RICE or mmu-miR146a in the small intestines of the mice fed the transgenic diets for 3 days. The intestinal contents were flushed out with 1 mL of PBS. *N* = 3, *error bars* represent SEM

When testing the bioavailability of the transgenic miRNAs using mice fed the plant diets, we concurrently analyzed the uptake of dietary miRNAs in circulation and the miRNA levels in the gut. The analysis of the gut content of the mice fed the transgenic plant diets revealed a difference in the surviving levels in the small intestines between MIR2911 and amiR-RICE or mmu-miR146a. On average, MIR2911 had a total abundance of about 238 fmol per mouse, while amiR-RICE only had about 2.7 fmol per mouse. mmu-miR146a was at a much higher level at around 38 fmol per mouse than amiR-RICE. However, a similar level was also detected in the chow-fed mice when they did not consume the transgenic plant material, suggesting that this mmu-miR146a was not derived from the diet (Fig. 4).

**Fig. 5:**
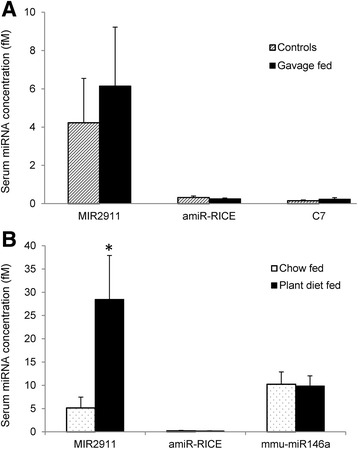
Bioavailability of transgenic miRNAs in mice. (**a**) qRT-PCR quantification of serum levels of miRNAs in mice gavage fed with a cocktail of 400 pmol each of MIR2911, amiR-RICE, and C7. Controls: mice gavage fed PBS. (**b**) qRT-PCR quantification of serum levels of miRNAs in mice fed the transgenic plant diets for 3 days. *N* = 5, *error bars* represent SEM. *p < 0.05 between plant diet-fed and chow-fed mice

In vitro and in vivo digestion assays demonstrated that amiR-RICE is at least as stable as MIR2911 (Fig. 3). However, when we analyzed sera from mice gavage fed 400 pmol of synthetic amiR-RICE, these miRNAs were not readily bioavailable (Fig. 5a). Mice fed diets supplemented with transgenic plant materials for 3 days also had negligible serum levels of the transgenic miRNAs. Furthermore, attempts to detect other plant miRNAs that have been reported by others to be bioavailable, albeit from other plant-based diets, were not successful (Additional file 3: Figure S3), However, the levels of MIR2911 in mice fed the plant diets (28.5 fM, or 1.3 × 10^7^ copies per mouse) were more than fivefold higher than animals receiving the chow-based diet (5.2 fM, or 2.3 × 10^6^ copies per mouse) (Fig. 5b). The enhanced levels of MIR2911 in the transgenic diet-fed mice served as a positive control for consumption of the diets and detection of plant-based RNAs in the mouse sera.

## Discussion

Disagreements are common but crucial in science; the nascent field of dietary sRNAs is certainly no stranger to these controversies [20]. Transgenic crops can express populations of miRNAs not found in nature, and in view of the conflicting reports inferring bioavailability of diet-derived miRNAs, the disagreements among scientists are profound because they impact the way agencies and agrobiotechnology regulate and use this technology.

The two plant-derived transgenic miRNAs tested here demonstrated modest digestive stability (Fig. 3) and were abundant in the transgenic plants (Fig. 1); however, they were not readily bioavailable when fed to healthy mice (Fig. 5). Our group has also been unable to reliably detect (<32 Ct) canonical plant-based miRNAs in the sera of mice consuming plant-based diets (Additional file 3: Figure S3; (28)). Future analysis should be performed with mice fed with the transgenic plants for longer intervals and detection measured in the intestine as well as in organs outside the gastro-intestinal tract.

A variety of plant-based miRNAs can be found within the plant cellular matrix and may be coupled with other plant molecules such as proteins and polysaccharides [33]. These alterations may provide a conduit for dietary plant-derived miRNA uptake [33]. Using in vitro experiments, miR168a in soybean and mir166 in rice demonstrate more resistance to degradation than miR168a from rice and mir166 from soybean [33]. There are no sequence differences between these miRNAs; thus, it has been proposed that plant-specific mechanisms afford varying levels of protection from degradation. If this is true, bioavailability tests in transgenic Arabidopsis lines may not equate to bioavailability in other transgenic plants. Plant-specific exosome-like nanoparticles (EPDENs) may also mediate interspecies communication [37], but it remains an open question if these effects are mediated by miRNAs. In milk, specific miRNAs appear to be encapsulated in exosomes conferring protection against degradation and facilitating uptake [18, 19]. These mechanisms may facilitate bioavailability of selected plant-based miRNAs, but with the two miRNAs tested here, they did not provide a conduit for absorption of the specific transgenic miRNAs tested here.

While these two transgenic miRNAs do not appear to be bioavailable in healthy mice, disease and nutritional status are important influences controlling consumer nutrient absorption [38]. A confluence of diet and health issues converges to influence uptake of plant-based genetic material [14, 21, 28]. Pharmacological regimes also facilitate the detection of gavage-fed miRNAs. Future work will focus on assaying bioavailability of GM-derived miRNAs under these more permissive conditions.

Preparation methods could enhance the bioavailability of sRNAs in plant-based diets [16, 23]. For traditional nutrients, various strategies, including thermal processing, soaking, and fermentation, aim to increase the physicochemical accessibility of the nutrients while decreasing the content of antinutrients [39]. Future work will need to be directed at how food processing and preparation practices impact the dietary sRNA quality of plant-based foods.

MIR2911 has several features that facilitate its bioavailability that do not appear to be characteristics of the miRNAs tested here: first, the GC-rich MIR2911 has a high digestive stability [16, 27]; secondly, a protein complex protects MIR2911 [27]; third, synthesis via rRNA degradation dramatically increases MIR2911 abundance post-harvest [31]. The difference in intestinal levels of MIR2911 and the transgenic miRNAs could be explained by the degradation of the ribosomal RNAs that generate increased levels of MIR2911 [31]. In order for transgenic miRNAs to become more bioavailable, strategies need to be deployed that co-opt some or all of these tactics. It is interesting that one of the two transgenic miRNAs tested, amiR-RICE, was found to have similar digestive stability to MIR2911 in the in vitro system, or when gavage fed to the mice, but was not bioavailable. We posit this could be caused by either selective transport by the gut or differential stability or metabolism within the cells or in circulation.

Low bioavailability of the plant-based miRNAs does not negate potential bioactivity. Curcumin, a plant-based product, exhibits poor systemic bioavailability but still has potent pharmacological effects [40]. Like curcumin, emerging studies demonstrate that femtomolar amounts of a specific miRNA altered the fate of a targeted cell [41]. The miRNAs released by cancer cells can act as hormones [42, 43]. Additionally, evidence suggests that plant sRNAs have therapeutic effects that are sequence independent [44], allowing speculation that it is the additive abundance of trace amounts of a variety of different plant-based sRNAs that confer biological activity. Low amounts of miRNAs may have biological functions that could revolutionize our concepts of plant-based bioactives.

## Conclusions

Studies continue to suggest cross-kingdom gene regulation by dietary miRNAs; meanwhile, the majority of work has questioned the validity of these reports [20, 24, 45, 46]. This pilot study suggests that the two transgenic miRNAs tested are not readily bioavailable to healthy consumers and therapeutic plant-based dietary miRNAs may need to focus on establishing conditions that allow miRNAs to overcome the obstacles hindering bioavailability.

## Additional files

Additional file 1: Figure S1. Standard curves of synthetic miRNAs. Standard curves for qRT-PCR analysis generated with serial dilutions of synthetic MIR2911, amiR-RICE, mmu-miR146a, or C7. (TIFF 762 kb)

Additional file 2: Figure S2. Quantification of mmu-miR146a from transgenic Arabidopsis lines. qRT-PCR quantification of mmu-miR146a expression from eight independent transgenic lines. The arrow indicates the line (tg-146) used for diet preparation. (TIFF 525 kb)

Additional file 3: Figure S3. Quantification of MIR172a and MIR168a in the sera of mice fed Arabidopsis diets. qRT-PCR quantification of plant MIR172a and MIR168a in the sera of mice fed either chow diet or Arabidopsis diets (tg-RICE and tg-146 combined data). Ct values normalized to the mouse endogenous Let-7d,g,i. (TIFF 637 kb)

## References

[1] James C (2010). A global overview of biotech (GM) crops: adoption, impact and future prospects. GM Crops.

[2] Sakakibara K, Saito K (2006). Review: genetically modified plants for the promotion of human health. Biotechnol Lett.

[3] Gartland KMA, Bruschi F, Dundar M, Gahan PB (2013). Progress towards the ‘Golden Age’ of biotechnology. Curr Opin Biotechnol.

[4] Kuiper HA, Kleter GA, Noteborn HP, Kok EJ (2001). Assessment of the food safety issues related to genetically modified foods. Plant J.

[5] Varzakas TH, Chryssochoidis G, Argyropoulos D (2007). Approaches in the risk assessment of genetically modified foods by the Hellenic Food Safety Authority. Food Chem Toxicol.

[6] Ramon M, Devos Y, Lanzoni A, Liu Y (2014). RNAi-based GM plants: food for thought for risk assessors. Plant Biotechnol J.

[7] Levine E, Zhang Z, Kuhlman T, Hwa T (2007). Quantitative characteristics of gene regulation by small RNA. PLoS Biol.

[8] Dickinson B, Zhang Y, Petrick JS, Heck G (2013). Lack of detectable oral bioavailability of plant microRNAs after feeding in mice. Nat Biotechnol.

[9] Witwer KW, McAlexander MA, Queen SE, Adams RJ (2013). Real-time quantitative PCR and droplet digital PCR for plant miRNAs in mammalian blood provide little evidence for general uptake of dietary miRNAs: limited evidence for general uptake of dietary plant xenomiRs. RNA Biol.

[10] Petrick JS, Brower-Toland B, Jackson AL, Kier LD (2013). Safety assessment of food and feed from biotechnology-derived crops employing RNA-mediated gene regulation to achieve desired traits: a scientific review. Regul Toxicol Pharmacol.

[11] Petrick JS, Moore WM, Heydens WF, Koch MS, Sherman JH (2015). A 28-day oral toxicity evaluation of small interfering RNAs and a long double-stranded RNA targeting vacuolar ATPase in mice. Regul Toxicol Pharmacol.

[12] Knudsen I, Poulsen M (2007). Comparative safety testing of genetically modified foods in a 90-day rat feeding study design allowing the distinction between primary and secondary effects of the new genetic event. Regul Toxicol Pharmacol.

[13] Snow JW, Hale AE, Isaacs SK, Baggish AL, Chan SY (2013). Ineffective delivery of diet-derived microRNAs to recipient animal organisms. RNA Biol.

[14] Yang J, Farmer LM, Agyekum AAA, Elbaz-Younes I, Hirschi KD (2015). Detection of an abundant plant-based small RNA in healthy consumers. PLoS One.

[15] Chin AR, Fong MY, Somlo G, Wu J*,* et al*.* Cross-kingdom inhibition of breast cancer growth by plant miR159. Cell Res. 2016;20(2):217–28. 10.1038/cr.2016.13 PMC4746606 26794868

[16] Zhou Z, Li X, Liu J, Dong L (2015). Honeysuckle-encoded atypical microRNA2911 directly targets influenza A viruses. Cell Res.

[17] Zhang L, Hou D, Chen X, Li D (2012). Exogenous plant MIR168a specifically targets mammalian LDLRAP1: evidence of cross-kingdom regulation by microRNA. Cell Res.

[18] Zempleni J, Baier SR, Hirschi KD (2015). Diet-responsive MicroRNAs are likely exogenous. J Biol Chem.

[19] Baier SR, Nguyen C, Xie F, Wood JR, Zempleni J (2014). MicroRNAs are absorbed in biologically meaningful amounts from nutritionally relevant doses of cow milk and affect gene expression in peripheral blood mononuclear cells, HEK-293 kidney cell cultures, and mouse livers. J Nutr.

[20] Witwer KW, Hirschi KD (2014). Transfer and functional consequences of dietary microRNAs in vertebrates: concepts in search of corroboration: negative results challenge the hypothesis that dietary xenomiRs cross the gut and regulate genes in ingesting vertebrates, but important questions persist. Bioessays.

[21] Yang J, Hirschi KD, Farmer LM (2015). Dietary RNAs: new stories regarding oral delivery. Nutrients.

[22] Mlotshwa S, Pruss GJ, MacArthur JL, Endres MW (2015). A novel chemopreventive strategy based on therapeutic microRNAs produced in plants. Cell Res.

[23] Pastrello C, Tsay M, McQuaid R, Abovsky M (2016). Circulating plant miRNAs can regulate human gene expression in vitro. Sci Rep.

[24] Witwer KW (2014). Diet-responsive mammalian miRNAs are likely endogenous. J Nutr.

[25] Bagci C, Allmer J (2016). One step forward, two steps back; xeno-microRNAs reported in breast milk are artifacts. PLoS One.

[26] Auerbach A, Vyas G, Li A, Halushka M, Witwer K (2016). Uptake of dietary milk miRNAs by adult humans: a validation study. F1000Res.

[27] Yang J, Hotz T, Broadnax L, Yarmarkovich M (2016). Anomalous uptake and circulatory characteristics of the plant-based small RNA MIR2911. Sci Rep.

[28] Yang J, Farmer LM, Agyekum AAA, Hirschi KD (2015). Detection of dietary plant-based small RNAs in animals. Cell Res.

[29] Zhang X, Yuan YR, Pei Y, Lin SS (2006). Cucumber mosaic virus-encoded 2b suppressor inhibits Arabidopsis Argonaute1 cleavage activity to counter plant defense. Genes Dev.

[30] Tsuzuki M, Takeda A, Watanabe Y (2014). Recovery of dicer-like 1-late flowering phenotype by miR172 expressed by the noncanonical DCL4-dependent biogenesis pathway. RNA.

[31] Yang et al. Mol Nutr Food Res. http://onlinelibrary.wiley.com/doi/10.1002/mnfr.201600974/abstract;jsessionid=123206D8B1EA5E15E9516E804C2927AC.f04t04.

[32] Chen X, Liang H, Guan D, Wang C (2013). A combination of Let-7d, Let-7g and Let-7i serves as a stable reference for normalization of serum microRNAs. PLoS One.

[33] Philip A, Ferro VA, Tate RJ (2015). Determination of the potential bioavailability of plant microRNAs using a simulated human digestion process. Mol Nutr Food Res.

[34] Minekus M, Alminger M, Alvito P, Ballance S (2014). A standardised static in vitro digestion method suitable for food—an international consensus. Food Funct.

[35] Huala E, Dickerman AW, Garcia-Hernandez M, Weems D (2001). The Arabidopsis Information Resource (TAIR): a comprehensive database and web-based information retrieval, analysis, and visualization system for a model plant. Nucleic Acids Res.

[36] Dai Y, Jia P, Fang Y, Liu H (2016). miR-146a is essential for lipopolysaccharide (LPS)-induced cross-tolerance against kidney ischemia/reperfusion injury in mice. Sci Rep.

[37] Mu J, Zhuang X, Wang Q, Jiang H (2014). Interspecies communication between plant and mouse gut host cells through edible plant derived exosome-like nanoparticles. Mol Nutr Food Res.

[38] Krehl WA (1962). Factors affecting utilization and requirements. Vitamins and minerals. Am J Clin Nutr.

[39] Hotz C, Gibson RS (2007). Traditional food-processing and preparation practices to enhance the bioavailability of micronutrients in plant-based diets. J Nutr.

[40] Schiborr C, Kocher A, Behnam D, Jandasek J (2014). The oral bioavailability of curcumin from micronized powder and liquid micelles is significantly increased in healthy humans and differs between sexes. Mol Nutr Food Res.

[41] Bryniarski K, Ptak W, Martin E, Nazimek K (2015). Free extracellular miRNA functionally targets cells by transfecting exosomes from their companion cells. PLoS One.

[42] Fabbri M, Paone A, Calore F, Galli R (2012). MicroRNAs bind to Toll-like receptors to induce prometastatic inflammatory response. Proc Natl Acad Sci U S A.

[43] Galli R, Paone A, Fabbri M, Zanesi N (2013). Toll-like receptor 3 (TLR3) activation induces microRNA-dependent reexpression of functional RARβ and tumor regression. Proc Natl Acad Sci U S A.

[44] Cavalieri D, Rizzetto L, Tocci N, Rivero D (2016). Plant microRNAs as novel immunomodulatory agents. Sci Rep.

[45] Title AC, Denzler R, Stoffel M (2015). Uptake and function studies of maternal milk-derived microRNAs. J Biol Chem.

[46] Chan SY, Snow JW. Uptake and impact of natural diet-derived small RNA in invertebrates: implications for ecology and agriculture. RNA Biol. 2016;14(4):402–14. 10.1080/15476286.2016.1248329 PMC5411125 27763816

