# Supplementary figures and images for: Bioavailability of transgenic microRNAs in genetically modified plants
Source: Genes Nutr. 2017 Jul 7;12:17. doi: 10.1186/s12263-017-0563-5 (PMC5831112; doi:10.1186/s12263-017-0563-5)

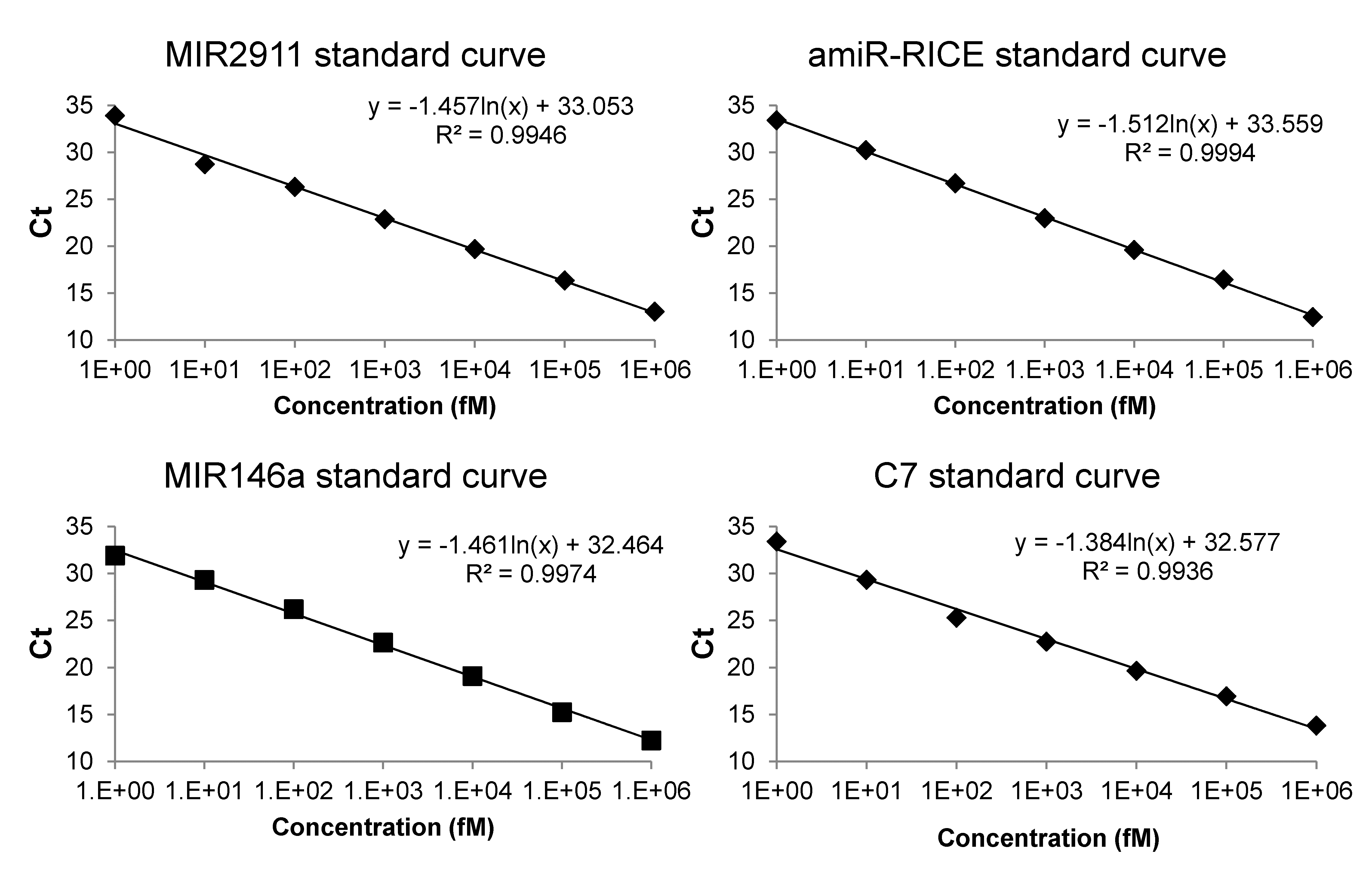

Supplement: Supplementary file 1 — Standard curves of synthetic miRNAs. Standard curves for qRT-PCR analysis generated with serial dilutions of synthetic MIR2911, amiR-RICE, mmu-miR146a, or C7. (TIFF 762 kb) [file 12263_2017_563_MOESM1_ESM.tiff]

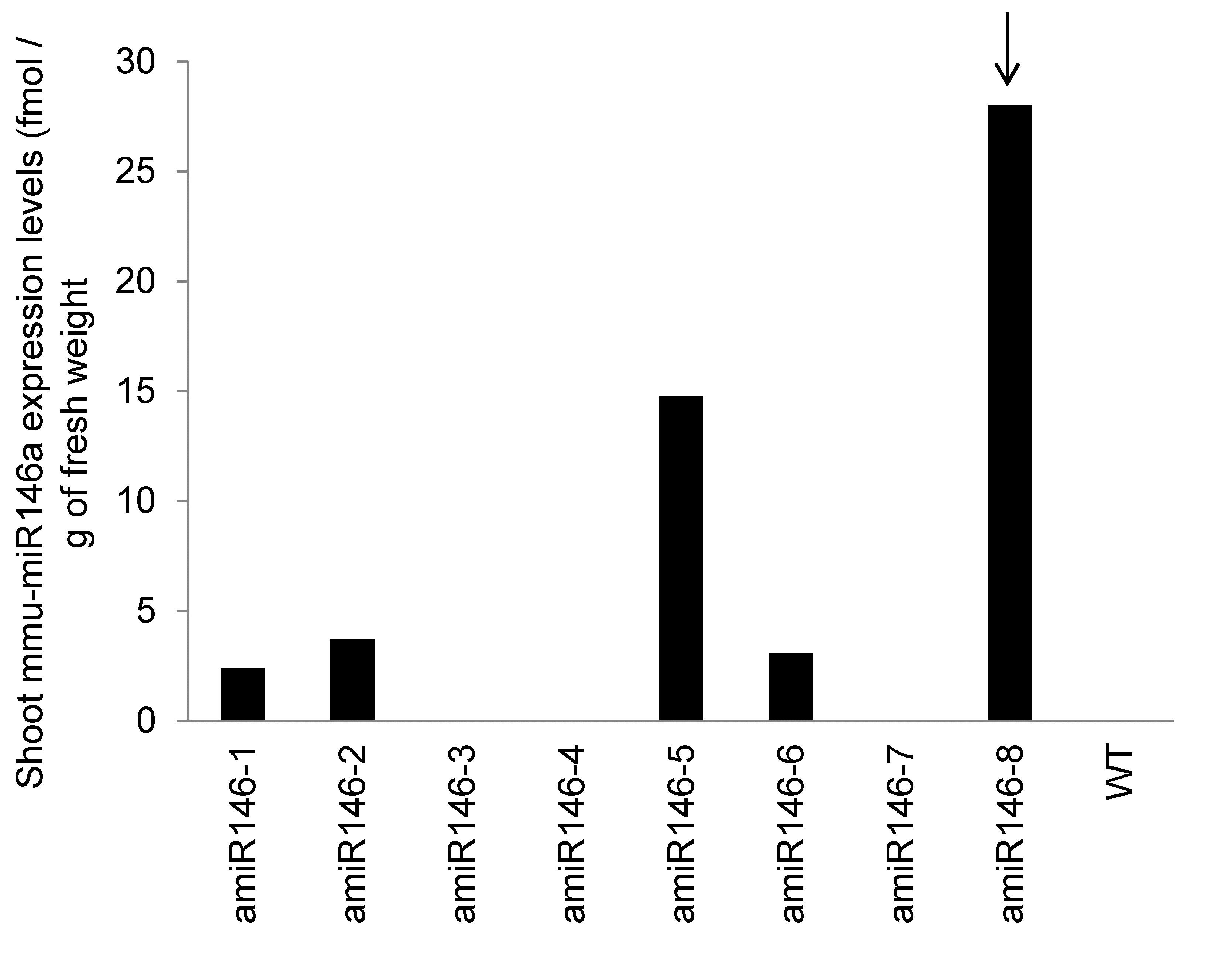

Supplement: Supplementary file 2 — Quantification of mmu-miR146a from transgenic Arabidopsis lines. qRT-PCR quantification of mmu-miR146a expression from eight independent transgenic lines. The arrow indicates the line (tg-146) used for diet preparation. (TIFF 525 kb) [file 12263_2017_563_MOESM2_ESM.tiff]

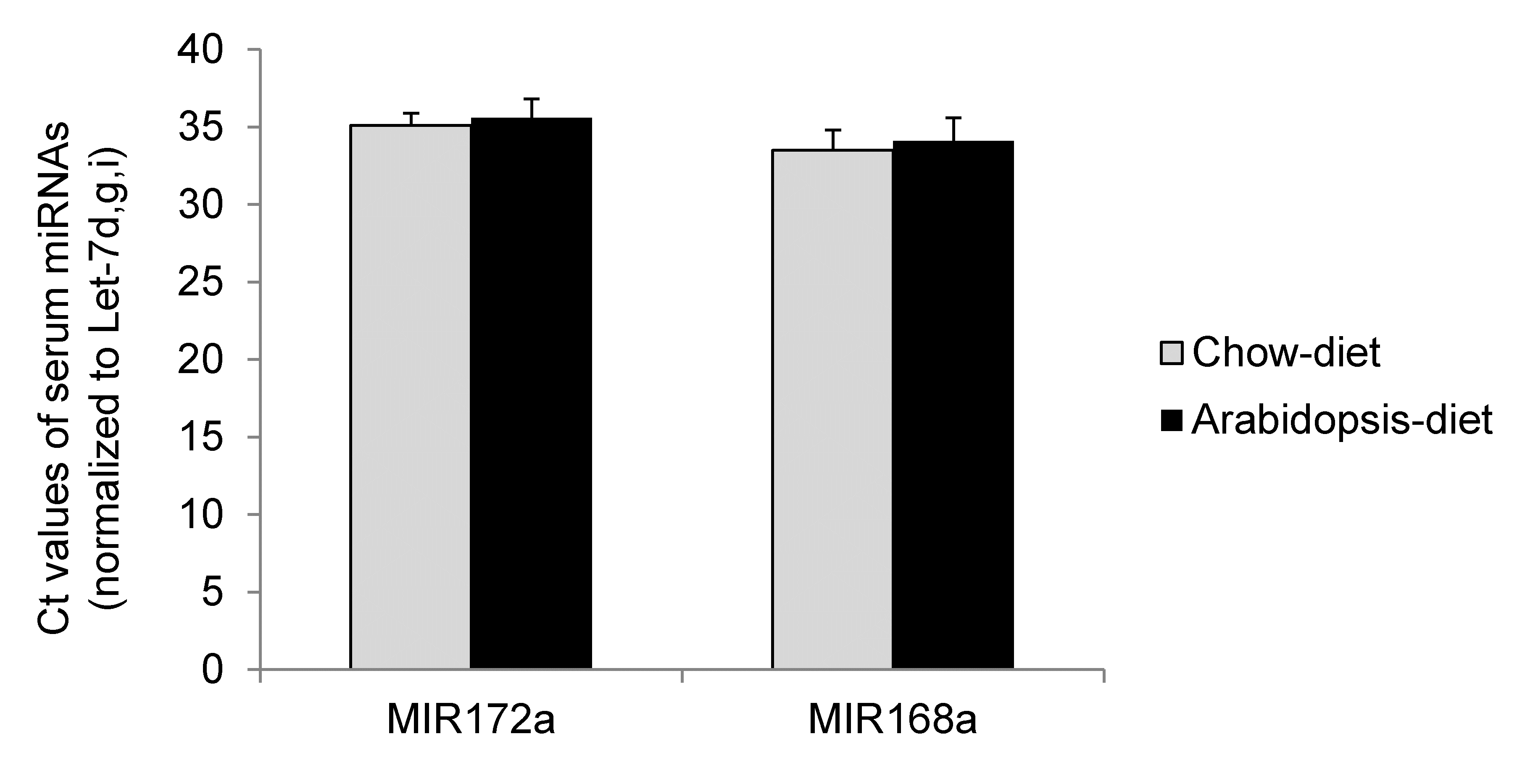

Supplement: Supplementary file 3 — Quantification of MIR172a and MIR168a in the sera of mice fed Arabidopsis diets. qRT-PCR quantification of plant MIR172a and MIR168a in the sera of mice fed either chow diet or Arabidopsis diets (tg-RICE and tg-146 combined data). Ct values normalized to the mouse endogenous Let-7d,g,i. (TIFF 637 kb) [file 12263_2017_563_MOESM3_ESM.tiff]
